# Morbidity and mortality in alcohol use disorder: the role of comorbid substance use disorder, age, sex, and the A1 allele of the Taq1A (rs1800497) polymorphism in the ANKK1 gene in an 18-year follow-up

**DOI:** 10.1093/alcalc/agag020

**Published:** 2026-04-05

**Authors:** Kerstin K Rauwolf, Ulf Berggren, Jan Balldin, Caroline Hasselgren Bune, Kristina J Berglund

**Affiliations:** Department of Pediatric Hematology and Oncology, Albert-Schweitzer-Campus 1, 48149 Muenster, University Hospital of Muenster, Germany; Institute of Neuroscience and Physiology, Department of Psychiatry and Neurochemistry, Sahlgrenska Academy, Medicinargatan 11, SE 413 90, Gothenburg, University of Gothenburg, Sweden; Institute of Neuroscience and Physiology, Department of Psychiatry and Neurochemistry, Sahlgrenska Academy, Medicinargatan 11, SE 413 90, Gothenburg, University of Gothenburg, Sweden; Department of Sociology and Work Science, Skanstorget 18, SE 411 22 Gothenburg, University of Gothenburg, Sweden; Department of Psychology, Haraldsgatan 1, SE 413 14 Gothenburg, University of Gothenburg, Sweden

**Keywords:** Alcohol Use Disorder, Substance Use Disorder, the A1 allele of the Taq1A polymorphism, mortality, age, gender

## Abstract

**Background and aims:**

The present study aimed to: (i) compare a patient group with solely alcohol use disorder (AUD) to a group with poly-substance use disorder (poly-SUD) regarding sociodemographic background, morbidity, mortality, and the prevalence of the A1 allele of the Taq1A polymorphism. (ii) Investigate whether gender, age, poly-SUD, and the prevalence of the A1 allele or interactions among these factors, are associated with mortality risk over an 18-year follow-up period.

**Methods:**

This study comprised 360 individuals treated for severe alcohol withdrawal symptoms in 1997 at a treatment unit in Sweden. Genotyping was performed during their hospital stay, and participants were followed annually for up to 18 years using data from Swedish registers.

**Results:**

Fifty-three percent of the participants had died over the 18 year period. Poly-SUD patients exhibited higher rates of psychiatric disorders, gastrointestinal diseases, and intoxication as the primary diagnosis. Patients with AUD exhibited a higher prevalence of cardiac diseases. Traumatic causes of death were more prevalent in the poly-SUD group, whereas somatic diseases were more common among individuals with AUD. Male sex and age were the strongest predictors of premature death among individuals with AUD. The A1 allele of the Taq1A polymorphism showed a borderline association with an increased hazard of death.

**Conclusions:**

Male sex and age are the strongest predictors of premature death. Patients with poly-SUD may represent a distinct subgroup with different comorbidities and causes of death. To determine whether there is a genetic vulnerability as indicated by the findings, research using larger samples with sufficient statistical power is needed.

## Teaser

**Short summary** To compare a patient group with AUD to a group with Poly-substance Use Disorder regarding sociodemographic background, morbidity, mortality, and the prevalence of the A1 allele of the Taq1A polymorphism in a 18-year follow-up. Male sex and age were the strongest predictors of premature death.

## Introduction

Harmful alcohol use is well-known to be associated with increased mortality, accounting for an estimated 5.3% of all deaths worldwide (WHO 2024). Furthermore, excessive alcohol consumption contributes to premature death and disability, particularly early in life. Among individuals aged 20–39 years, ~13.5% of total deaths are attributed to excessive alcohol consumption (WHO 2024). A recent study from the USA estimated that 1 in 8 total deaths among US adults aged 20–64 years were attributable to excessive alcohol use, with as many as 1 in 5 deaths in the 20–49-year age group (Esser et al. 2014). In the Nordic countries (Denmark, Finland, and Sweden), which have relatively restrictive alcohol control policies compared to other nations, individuals hospitalized for alcohol use disorder (AUD) were found to have an average life expectancy of 47–53 years for men and 50–58 years for women (Westman et al. 2015). Furthermore, it was estimated that they died 24–28 years earlier than individuals in the general population (Westman et al. 2015). It thus appears that alcohol-related deaths can occur early in life, thereby significantly reducing life expectancy.

The increased mortality rate among individuals with harmful alcohol use or AUD has been reported to be associated with several factors, including male sex, older age, unemployment, accidents, and alcohol-related somatic diseases (Feuerlein et al. 1994, Timko et al. 2006, Pavarin et al. 2015, Holst et al. 2017, Hjemsæter et al. 2019). In this context, it should be noted that there is a lack of studies on mortality in heterogeneous clinical samples, which often include individuals with AUD alone as well as those with poly-substance use disorder (poly-SUD) (Hjemsæter et al. 2019). In a 19-year prospective cohort study by Hjemsaeter et al. (Hjemsæter et al. 2019), it was found that individuals with AUD alone died more frequently from somatic diseases, whereas individuals with poly-SUD died more frequently from overdose. Furthermore, little is known about the contribution of specific genes to the mortality in individuals with AUD beside the exception of an earlier study of our group, which included the present group of patients with AUD and a history of inpatient treatment (Berggren et al. 2010). Thus, in the study by Berggren et al. (Berggren et al. 2010) we found that at a 10-year follow-up, individuals who were carriers of the A1 allele of the Taq1A polymorphism had an increased standardized mortality rate of ~40% compared to non-carriers. At an 18-year follow-up of this patient group, we found that carriers of the A1 allele of the Taq1A polymorphism had a shorter survival time, even when controlling for the influence of age and gender (Balldin et al. 2018).

The aim of the present study was:

- (i) Compare a patient group with solely AUD to a group with poly-SUD regarding sociodemographic background, morbidity, mortality, and the prevalence of the A1 allele of the Taq1A polymorphism.
- (ii) Investigate whether gender, age, poly-SUD, and the prevalence of the A1 allele of the Taq1A polymorphism, or interactions among these factors, are associated with mortality risk over an 18-year follow-up period.

## Materials and methods

### Patients

The study sample comprised adult patients (aged 20–76 years) who were admitted in 1997 to a specialized inpatient unit for severe alcohol and drug withdrawal at a psychiatric clinic within a university hospital in Western Sweden (*n* = 360). All participants met the criteria for AUD (American Psychiatric Association 2022) (formerly diagnosed as alcohol dependent/alcohol abuse; American Psychiatric Association 1994) at the time of admission although some had been diagnosed during previous hospital stays. During this inpatient stay, the patients were also genotyped for the A1 allele of the Taq1A polymorphism of the ankyrin repeat and kinase domain containing 1 (ANKK1) locus. For further details on the genotyping procedure, see Berggren et al. (Berggren et al. 2006, Berggren et al. 2010) and Balldin et al. (Balldin et al. 2018). The patients were provided with information about the study and gave their consent to participate. The Regional Ethical Review Board in Gothenburg approved the study (No. 094-14), including the data linkage.

### Description of data collection

Information on diagnoses, sex, age, and genotype was obtained from hospital records during the inpatient treatment episode in 1997. We have also received patient records spanning from 1997 to 2015, enabling us to continue investigation of the medical conditions and diagnoses that emerged during this period. Other background variables, such as education, marital status, income, occupation, and causes of death, were obtained from Swedish registers (Cause of Death Registry, Socioeconomic Register). These different registers were linked using the unique 10-digit personal identification numbers assigned to all individuals born in Sweden or those who had immigrated to Sweden. However, there is a lack of personal identification numbers for 15 individuals, resulting in the inability to link the different registers. Therefore, 360 individuals were included. In this study, background data were obtained from the Socioeconomic Register (1997), and mortality data were obtained from the Cause of Death Registry for the period 2004–2015.

### Data processing and statistical analyses

We reviewed all the available medical records for each individual between 1997 and 2015, examining whether, in addition to an AUD diagnosis, there were also diagnoses of other substances (opioids, cannabinoids, sedatives, hypnotics, and stimulants). A diagnosis of substance use disorder was considered as belonging to the poly-SUD group. Two researchers independently reviewed the records to ensure accurate classification of the participants. Similarly, based on the records, we also identified the various other medical and psychiatric diagnoses that the participants had.

When comparing background data, as well as causes of death and morbidity, between individuals with AUD alone and those with poly-SUD, we used standard statistical methods (t-tests and chi-square tests). We examined effect sizes in the pairwise comparisons (Cohen’s d or φ [phi-coefficient]).

Cox proportional hazards regression models were used to examine the effects of age, sex, poly-SUD, and the A1 allele of the Taq1A polymorphism on survival, including potential interaction effects. Time at risk was calculated in years from the year of admission to the alcohol and drug treatment unit (1997) to the year of death or the end of follow-up in 2015 (maximum 18 years). As exact dates and months of death were unavailable for some participants, survival time was measured in whole years. For deaths occurring in 1997 (*N* = 2), a small positive survival time was assigned to enable inclusion in the Cox regression model. Participants who were alive at the end of follow-up were censored at 18 years. Model fit was compared using likelihood ratio tests for nested models (Allison 2010). As part of the interaction analyses, we present plots of predicted survival for relevant combinations of covariates, as raw estimates from interaction models should not be interpreted as unconditional marginal effects (Brambor et al. 2006). The proportional hazards assumption was assessed for all models, and no evidence of violation was detected (Allison 2010). The significance level was set at *P* < .05. Descriptive analyses were conducted using SPSS version 28 and interaction models with associated plots were computed using R version 4.5.0 (R Core Team, 2025).

## Results

### Sociodemographic and psychiatric characteristics of the AUD and poly-SUD groups

The study sample 1997 comprised 360 individuals. The left side of Table 1 describes the study population, divided into those with solely AUD and those with poly-SUD. There were 226 participants (63%) with AUD and 134 (37%) with poly-SUD. As depicted in Table 1, there was an even distribution of gender between the two groups. However, it should be noted that across the entire group, there were significantly more men (81%) than women (19%). Significantly more individuals in the AUD group were older than 60 years (15%) compared to the poly-SUD group (3%). Conversably, there were more younger individuals (20–40 years) in the poly-SUD group (42%) compared to those with solely AUD (16%) χ^2^(2, 360) = 36.11, *P <* .001, φ *=* 0.317. Significantly more individuals in the poly-SUD group lived in single households (75% vs. 62%; χ^2^(1, 360) = 6.44, *P =* .011, φ *=* 0.134), had significantly lower annual incomes (*t*(355) = 3.420, *d* = 0.37, *P* < .001), and were less likely to be employed (32% vs. 48%; χ^2^(1, 359) = 10.08, *P =* .002, φ *=* 0.168). In the poly-SUD group, there were higher percentages of individuals with affective disorders (44% vs. 28%; χ^2^(1, 360) = 9.80, *P =* .002, φ *=* 0.165), anxiety disorders (52% vs. 24%; χ^2^(1, 360) = 28.90, *P <* .001, φ *=* 0.283), personality disorders (18% vs. 2%; χ^2^(1, 360) = 30.55, *P <* .001, φ *=* 0.291), psychotic disorder (9% vs. 3%; χ^2^(1, 360) = 5.77, *P =* .016, φ *=* 0.127), dementia disorders (11% vs. 4%; χ^2^(1, 360) = 5.97, *P =* .015, φ *=* 0.129), and total numbers of psychiatric disorders (*t*(358) = −7.87, *d* = 0.86, *P* < .001).

**Table 1:** Descriptive characteristics of study population (left side of the table; *n* = 360) and deceased study population between 1998–2015 (right side of the table *n* = 193)

Baseline characteristics	Total *n* = 360	AUD *n* = 226 (62.8%)	Poly-SUD *n* = 134 (37.2%)	*P*	Effect size	Deceased AUD *n* = 126	Deceased poly-SUD *n* = 67	*P*	Effect size
Male sex (%)	292 (81.1%)	186 (82.3%)	106 (79.1%)	*ns*	0.062	113 (89.7%)	56 (83.6%)	*ns*	0.116
Age-groups									
20–40 years 41—60 years > 60 years	92 (25.6%) 230 (63.9%) 38 (10.6%)	36 (15.9%) 156 (69.0% 34 (15.0%)	56 (41.8%) 74 (55.2%) 4 (3.0%)	<.001	0.317	11 (8.7%) 89 (70.6%) 26 (20.6%)	26 (38.8%) 39 (58.2%) 2 (3.0%)	< .001	0.401
Single household (yes %)	242 (67.2%)	141 (62.4%)	101 (75.3%)	.011	0.134	77 (61.1%)	50 (74.6%)	*ns*	0.137
Upper secondary school (yes %)	185 (51.4%)	116 (51.3%)	69 (51.5%)	ns	0.011	63 (50.0%)	31 (46.2%)	*ns*	0.011
Employed (yes %)	151 (41.9%)	109 (48.2%)	43 (32.1%)	.006	0.151	53 (42.1%)	20 (29.9%)	*ns*	0.118
Income 1997 (Swedish crones)	113 248 ± 48 697	119 228 ± 54 118	101 382 ± 37 347	<.001	0.370	115 309 ± 54 950	101 585 ± 33 019	*ns*	0.265
The A1 allele A1 + A1 -	140 (38.9%) 219 (60.8%)	89 (39.3%) 136 (60.2%)	51 (38.1%) 83 (61.9%)	ns	0.002	51 (40.5%) 75 (59.5%)	32 (47.8%) 35 (52.2%)	ns	0.070
Years of survival	12.53 ± 5.59	12.12 ± 5.78	13.24 ± 5.18	ns	0.176	8.41 ± 5.19	9.19 ± 4.91	ns	0.190
Affective disorders	122 (33.9%)	63 (27.8%)	59 (44.0%)	.003	0.165	27 (21.4%)	22 (32.8%)	ns	0.125
Anxiety disorders	125 (34.7%)	55 (24.3%)	70 (52.2%)	<.001	0.283	25 (19.8%)	25 (37.3%)	.014	0.190
Personality disoders	28 (7.8%)	4 (1.8%)	24 (17.9%)	<.001	0.291	0 (0%)	9 (13.4%)	<.001	0.303
Dementia disorders	25 (6.9%)	10 (4.4%)	15 (11.2%)	.015	0.129	7 (5.6%)	9 (13.4%)	ns	0.136
Psychotic disorders	19 (5.3%)	7 (3.1%)	12 (9.0%)	.016	0.127	2 (1.6%)	5 (7.5%)	ns	0.150
Number of psychiatric symptom disorders (not SUD)	0.95 ± 1.05	0.64 ± 0.82	1.47 ± 1.19	<.001	0.858	0.50 ± 0.69	1.09 ± 1.12	<.001	0.680

### Psychiatric characteristics of deceased participants

We conducted comparisons between individuals who died between 1997 and 2015, comparing those with AUD and poly-SUD; see Table 1, right side. Significantly more younger individuals with poly-SUD (20–40 years) had died compared to those with solely AUD (39% vs. 9%; χ^2^(2, 193) = 31.05, *P <* .001, φ *=* 0.401). Significantly more individuals in the poly-SUD group with a diagnosis of personality disorder had died compared to those with solely AUD and a personality disorder (13% vs. 0%; χ^2^(1, 193) = 17.75 *P <* .001, φ *=* 0.303). Significantly more individuals in the poly-SUD group with a diagnosis of anxiety disorder had died compared to those with solely AUD and an anxiety disorder (37% vs. 20%; χ^2^(1, 193) = 6.96 *P =* .014, φ *=* 0.190). Individuals with poly-SUD who had died also had significantly more psychiatric diagnoses compared to those with solely AUD *t*(191) = −4.51, *d* = 0.68, *P* < .001.

### Main diagnosis of morbidity

We examined whether morbidities differed between AUD and poly-SUD patients. Significant differences were found (χ^2^(6, *N* = 358) = 21.24, *P* = .002, φ = 0.244). Gastrointestinal diseases were more common among poly-SUD patients (31.6% vs. 18.2%), whereas cardiac diseases were more common among AUD patients (16.4% vs. 5.3%) (see Table 2).

**Table 2:** Main diagnosis of morbidity. Data from the Swedish Patient register 1998–2015

Main diagnosis	Total *n* = 358	AUD *n* = 225	Poly-SUD *n* = 133	*P*	Effect size
Alcohol dependence	127 (35.5%)	85 (37.8%)	42 (31.6%)	.002	0.244
Cancer diseases	21 (5.9%)	14 (6.2%)	7 (5.3%)		
Cardiac diseases	44 (12.3%	37 (16.4%)	7 (5.3%)		
Intoxication	28 (7.8%)	12 (5.3%)	16 (12.0%)		
Gastrointestinal diseases	83 (23.2%)	41 (18.2%)	42 (31.6%)		
Neurological diseases	26 (7.3%)	16 (7.1%)	10 (7.6%)		
Other (infection, external causes/accident/pulmo)	29 (8.1%)	20 (8.9%)	9 (6.8%)		

### Principal cause of death

Between 1997 and 2015, 193 of the 360 individuals had died. The mortality rate over the 18-year follow-up period was 53.6%. The overall mean age at death was 58.4 ± 11.6 years, and it differed significantly between the two groups (AUD: 61.28 ± 10.75 years; poly-SUD: 52.97 ± 11.12 years; *t*(191) = 5.05, *d* = 0.76, *P* < .001). Table 3 presents the data we have from the causes of death register between 2004 and 2015, categorized by individuals with solely AUD and those with poly-SUD diagnoses (*n* = 121; 72 causes of death were missing). Traumatic deaths (overdoses, intoxication, suicide or accidents) were more prevalent among those with poly-SUD (39.0% vs. 14.7%, χ^2^(1, 121) = 6.76, *P =* .009, φ *=* 0.257). Regarding other causes of deaths, no significant differences were found between the two groups. For more details, see Table 3.

**Table 3:** Principal cause of death. Data from the Swedish Death Registry 2004—2015 (*n* = 121)

	Cause of death Total *n* = 121	Cause of death AUD *n* = 75	Cause of death poly-SUD *n* = 46	*P*	Effect size
**Somatic diseases (*n*; %)** Cancer disease (*n*) Heart/coronary disease (*n*) Alcohol, liver disease (*n*) Lung disease (*n*) Other (*n*)	61 (50.4%) 8 24 9 5 15	42 (56.0%) 6 20 4 3 9	19 (41.3%) 2 4 5 2 6	ns	−0.143
**Traumatic death (*n*; %)** Accidents Overdose/intoxication/suicide	28 (23.1%) 11 17	11 (14.7%) 8 3	17 (37.0%) 3 14	.009	0.257
**Alcohol dependence (*n*; %)**	18 (14.9%)	12 (16.0%)	6 (13.0%)	ns	−0.040
**Undetermined cause of death (*n*; %)**	14 (11.6%)	10 (13.3%)	4 (8.7%)	*ns*	−0.070

### Cox regression analysis—base model and interaction of age, gender, the A1 allele of the Taq1A polymorphism and poly-SUD associated with years of survival

In the base model without interaction terms, male sex was significantly associated with increased mortality risk (HR = 1.91, 95% CI [1.24–2.97], *P* = .004), and older age was strongly related to shorter survival time (HR = 1.03, 95% CI [1.02–1.04], *P* < .001). Neither poly-SUD (HR = 1.07, 95% CI [0.78–1.47], *P* = .689) nor the A1 allele of the Taq1A polymorphism (A1+) (HR = 1.32, 95% CI [0.99–1.76], *P* = .050) showed independent effects, although the latter displayed a borderline association with increased hazard of death.

**Figure 1:**
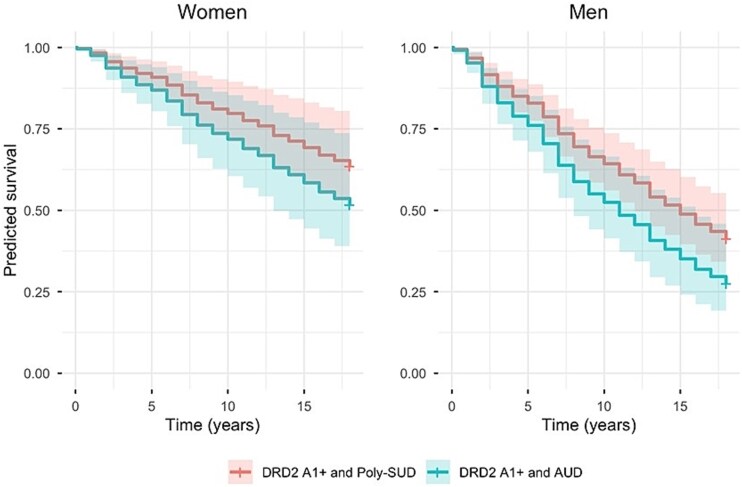
Plots of predicted survival based on Model 3. The figures display results for men and women separately.

Adding the interaction between sex and age (Model 2) did not significantly improve model fit (LR χ^2^(1) = 0.47, *P* = .491), indicating that the effect of age on survival did not differ between men and women. In contrast, when testing the interaction between poly-SUD and the A1 allele of the Taq1A polymorphism (A1+) (Model 3), a trend towards significance in model fit was observed (LR χ^2^(1) = 3.36, *P* = .067), suggesting a possible synergistic effect of genetic and poly-SUD on mortality. However, this effect was not significant (Table 4). Plots of predicted survival based on this model (Fig. 1) demonstrate that, in both men and women, the A1 allele of the Taq1A polymorphism (A1+) with poly-SUD exhibit consistently lower predicted survival relative to those with AUD only. However, the confidence intervals overlap throughout the follow-up period, implying that this difference could not be confirmed statistically in the current study.

**Table 4:** Tests of base model and interaction models (Cox regression, significant estimates in bold)

Model	HR	95% CI	*P*-value	*N*	Log-likelihood	LR χ^2^ (df)	*P*-value
1: Base model^d^, no interaction				358	−1048.43	–	–
Sex^a^	**1.91**	1.24–2.97	.004				
Age	**1.03**	1.02–1.04	<.001				
Poly-SUD^b^	1.07	0.78–1.47	.689				
A1+^c^	1.32	0.99–1.76	.055				
2: Interaction sex ^*^ age				358	−1048.24	0.39 (1)	.533
Sex^a^	0.90	0.08–9.98	.930				
Age	1.01	0.97–1.06	.558				
Poly-SUD^b^	1.08	0.78–1.49	.640				
A1+^c^	1.32	0.99–1.76	.058				
Sex ^*^ age	1.02	0.97–1.07	.535				
3: Interaction poly-SUD ^*^ A1+				358	−1046.76	3.36 (1)	.067
Sex^a^	**1.95**	1.25–3.02	.002				
Age	**1.03**	1.01–1.04	<.001				
Poly-SUD^b^	.83	0.55–1.27	.395				
A1+^c^	1.09	0.76–1.56	.641				
Poly-SUD ^*^ A1+	1.75	0.96–3.19	.067				
4: Interactions sex ^*^ age and poly-SUD ^*^ A1+				358	−1046.54	3.79 (2)	.151
Sex^a^	0.88	0.08–9.57	.922				
Age	1.01	0.97–1.06	.588				
Poly-SUD^b^	0.84	0.55–1.28	.424				
A1+^c^	1.08	0.76–1.55	.658				
Sex ^*^ age	1.02	0.97–1.07	.513				
Poly-SUD ^*^ A1+	1.76	0.97–3.20	.065				

^a^Ref = Woman.

^b^Ref = AUD.

^c^Ref = A1−.

^d^All comparisons are relative to Model 1.

Finally, a combined model including both interaction terms (Model 4) did not significantly improve overall model fit compared to the base model (LR χ^2^(2) = 3.79, *P* = .151).

## Discussion

The present findings highlight the importance of demographic, clinical, and biological factors in predicting premature mortality among individuals with AUD. Consistent with previous research, male sex and age emerged as robust predictors of reduced survival, reflecting well-documented gender differences in health behaviors, treatment engagement, and medical comorbidities associated with alcohol-related mortality (Feuerlein et al. 1994, Timko et al. 2006, Pavarin et al. 2015). In contrast, neither poly-SUD nor the A1 allele of the Taq1A polymorphism showed an independent association with mortality, although the genetic effect showed a trend towards significance, suggesting that Taq1A genetic variation may modestly contribute to adverse outcomes in AUD. Patients with poly-SUD died earlier compared to AUD alone. Deciphering the underlying causes of death during the 18-year follow-up, the primary causes of death in the AUD alone cohort were somatic diseases. In the poly-SUD cohort beside the somatic diseases, death was also caused by intoxication. But not only the live-expectancy in poly-SUD cohort was decreased compared to AUD alone, also the morbidity in these patients differed from the AUD alone cohort. Patients with poly-SUD had more often diseases of the gastrointestinal tract and intoxication while the risk for cardiac involvement was decreased compared to patients with AUD alone. This is in line with a Norwegian study by (Hjemsaeter et al. 2019) in which they emphasized the clinical implications of these different causes of death as well as morbidity because they require different preventive approaches. Patients with long-lasting AUD need better access to screening and treatment to prevent pre-mature death because of their somatic diseases. Interestingly, screening and treatment of their somatic disease is less systematic and effective compared to overdose prevention. Several large-scale strategies for decreasing death by overdose exist e.g. opioid maintenance treatment (Degenhardt et al. 2011). This study is a follow-up of an earlier study by us (Berggren et al. 2010) in which we found an increased mortality rate in carriers of the A1 allele of the Taq1A polymorphism during a follow-up period of 10 years.

In the present analysis, a trend towards an interaction between poly-SUD and the A1 allele of the Taq1A polymorphism was observed; however, this interaction did not reach statistical significance and confidence intervals overlapped. Consequently, this finding should be interpreted with caution and cannot be taken as evidence of a synergistic or amplifying effect.

Although the direction of the interaction is consistent with previous literature linking the A1 allele of the Taq1A polymorphism to traits such as impulsivity, altered reinforcement processing, and vulnerability to multiple substance use disorders (Noble 2003), the current results are exploratory in nature. The absence of statistical significance precludes conclusions regarding clinical relevance or predictive utility. Rather, these findings should be regarded as hypothesis-generating and highlight the need for larger, adequately powered studies to further examine potential gene–environment and gene–behavior interactions influencing long-term mortality in substance use populations.

From the results in this study, it may be estimated that the A1 allele of the Taq1A polymorphism shorten years of survival in alcohol-dependent individuals with about 10% during an 18-years follow-up period. This observation is very similar to that of male gender, which also shortens years of survival with 1.6 years. The combination of the A1 allele of the Taq1A polymorphism and male sex resulted in the greatest reduction in years of survival, followed by the combination of the A1 allele and female sex. The reasons why individuals with AUD and the A1 allele of the Taq1A polymorphism may exhibit shortened survival remain unclear. Importantly, the A1 allele of the Taq1A polymorphism is not located within the DRD2 gene itself but within the adjacent ANKK1 gene and therefore cannot be interpreted as reflecting direct genetic variation of the dopamine D2 receptor. Any associations observed between Taq1A1 carrier status and clinical outcomes must thus be considered indirect, potentially reflecting linkage or regulatory effects rather than receptor structure or expression per se (Hill et al. 2008).

Previous studies have reported that carriers of the A1 allele of the Taq1A polymorphism show an earlier onset of problematic drinking and higher levels of alcohol consumption (Connor et al. 2002, Connor et al. 2008, Panduro et al. 2017). Earlier work also described associations between the A1 allele of the Taq1A polymorphism and reduced dopamine D2 receptor availability (Gluskin & Mickey., 2016); however, the biological mechanisms underlying this relationship remain incompletely understood and should not be interpreted as evidence of a direct DRD2 effect. Hypotheses linking the A1 allele of the Taq1A polymorphism to altered reward processing or compensatory alcohol use through dopaminergic mechanisms therefore remain speculative. In addition to reward-related processes, cognitive and behavioral factors may also be relevant. Experimental studies in healthy individuals have suggested that carriers of the A1 allele of the Taq1A polymorphism may exhibit subtle impairments in cognitive flexibility and learning, including poorer reversal learning (Jocham et al. 2009), which could theoretically influence the ability to regulate or reduce alcohol consumption (Kumar et al. 2024). However, such interpretations are indirect and cannot be confirmed by the present data.

Accordingly, findings related to the A1 allele of the Taq1A polymorphism in the current study should be regarded as exploratory. While recent meta-analytic evidence suggests that the Taq1A1 locus is associated with susceptibility to addictive disorders across substances, effect sizes are small and functional significance remains uncertain (Kumar et al. 2024). Our results do not allow conclusions regarding specific neurobiological mechanisms or clinical implications but rather contribute to the broader literature examining genetic loci potentially associated with vulnerability to substance use disorders and their long-term outcomes.

There are some limitations in the present study that ought to be mentioned. The cause of death from AUD is not captured solely by genotype, gender and age, and the picture is far more complex. For example, the individuals in this study were not assessed for tobacco use and it cannot be ruled out that the difference in mortality rate between the groups can be due to difference in tobacco use. Furthermore, the increased mortality rate in the carriers of the A1 allele of the Taq1A polymorphism can be due to other factors. Another limitation of the data collection is that individuals with poly-SUD received their diagnosis at some point during the follow-up period. This may imply that some participants had a dual diagnosis for a shorter period, while others had it for a longer period during the study period. We nevertheless chose to classify the variable in this way, as there is substantial uncertainty for each individual regarding when their problems became severe enough to meet diagnostic criteria. Some individuals were likely undiagnosed for several years despite having poly-SUD, whereas others were identified earlier.

Despite these limitations, our findings suggest that while demographic factors remain the strongest predictors of survival, biological, and behavioral vulnerabilities—such as Taq1A genetic variation combined with polysubstance use—may have additive or interactive effects on long-term outcomes. Future studies with larger samples and longitudinal genetic data are warranted to clarify these relationships and to identify individuals at highest risk for premature death in the context of substance use disorders.

## Data Availability

The datasets used and/or analyzed during the current study are available from the corresponding author on reasonable request.
